# Prognostic nutritional index is associated with severe postoperative complications after neoadjuvant immunochemotherapy for esophageal squamous cell carcinoma: a retrospective cohort study with threshold effect analysis

**DOI:** 10.1186/s13019-026-04511-0

**Published:** 2026-07-19

**Authors:** Qiang Zhang, Qiuxia Liu, Zongxiang Lin, Pengfei Chen

**Affiliations:** Department of Thoracic and Cardiovascular Surgery, Affiliated Hospital of Putian University, No. 999, Dongzhen East Road, Licheng District, Putian City, 351100 Fujian Province China

**Keywords:** Esophageal squamous cell carcinoma, Neoadjuvant immunochemotherapy, Prognostic nutritional index, Severe Postoperative complications, Threshold effect, Pathological complete response

## Abstract

**Background:**

Neoadjuvant immunochemotherapy (nICT) followed by esophagectomy has become the standard treatment for locally advanced esophageal squamous cell carcinoma (LA–ESCC). However, severe postoperative complications remain a significant clinical concern. The prognostic nutritional index (PNI), reflecting both nutritional and inflammatory status, may identify patients at elevated risk for complications. This study aimed to investigate the dose–response relationship between preoperative PNI and severe postoperative complications, identify potential thresholds for risk stratification, and explore heterogeneity by pathological response.

**Methods:**

This retrospective cohort study included 134 consecutive patients with LA–ESCC who received nICT followed by radical esophagectomy. Preoperative PNI was calculated as serum albumin (g/L) + 5 × total lymphocyte count (×10⁹/L). The primary outcome was severe postoperative complications (SPCs). Multivariable logistic regression, restricted cubic spline analysis, threshold effect analysis, and subgroup analyses were performed to evaluate the independent association of PNI and explore nonlinear relationships.

**Results:**

SPCs occurred in 29 patients (21.6%). Multivariable analysis demonstrated that preoperative PNI was independently associated with SPCs (adjusted OR from the primary parsimonious model = 0.86, 95%CI: 0.78–0.96, *P* = 0.006). When PNI was categorized into quartiles, compared with Q1, the adjusted ORs for Q2, Q3, and Q4 were 0.22 (95% CI: 0.06–0.75, *P* = 0.016), 0.11 (95% CI: 0.03–0.46, *P* = 0.003), and 0.29 (95% CI: 0.08–1.06, *P* = 0.061), respectively, with a significant trend across quartiles (P for trend = 0.016). Restricted cubic spline analysis revealed a nonlinear “L–shaped” relationship between PNI and SPCs (P for non–linearity = 0.045). Threshold effect analysis identified 50.00 as the inflection point: below this threshold, each unit increase in PNI was associated with a 25% reduction in SPC risk (OR = 0.75, 95% CI: 0.594–0.936, *P* = 0.011); above this threshold, no significant association was observed (OR = 1.09, 95% CI: 0.872–1.372, *P* = 0.438). Subgroup analysis revealed significant heterogeneity by pathological response (P for interaction = 0.011): the protective effect of higher PNI was pronounced in non–pCR patients (OR = 0.80, 95% CI: 0.69–0.92) but absent in patients achieving pCR (OR = 1.06, 95% CI: 0.88–1.28).

**Conclusions:**

Preoperative PNI is independently associated with severe postoperative complications in LA–ESCC patients receiving nICT, exhibiting a nonlinear “L–shaped” relationship with a threshold of 50.00. The observed interaction with pCR status is exploratory and hypothesis–generating. Future prospective studies are needed to confirm these findings before clinical implementation.

## Introduction

Esophageal cancer ranks seventh in incidence and sixth in cancer–related mortality worldwide; in China, esophageal squamous cell carcinoma (ESCC) accounts for > 90% of cases and is the fourth leading cause of cancer death [1–3]. Most patients present with locally advanced disease, necessitating multimodal treatment [4].

For locally advanced ESCC (LA–ESCC), neoadjuvant therapy followed by surgery has emerged as a promising treatment strategy. The CROSS and NEOCRTEC5010 trials established neoadjuvant chemoradiotherapy in Western and Chinese populations [5, 6]. More recently, neoadjuvant immunochemotherapy (nICT) has shown promising results, with improved pathological complete response (pCR) and disease–free survival compared with chemotherapy alone in advanced esophageal cancer [7]; nICT is increasingly investigated in LA–ESCC [8, 9].

Despite these advances, severe postoperative complications following esophagectomy remain a major challenge, affecting 20–70% of patients and contributing to prolonged hospitalization, higher costs, delayed adjuvant treatment, and worse long–term survival [10–12]. Reliable preoperative risk stratification is urgently needed.

The prognostic nutritional index (PNI), originally developed by Onodera et al. [13], is a simple index calculated from serum albumin and peripheral lymphocyte count. It reflects both nutritional and immunological status, which are closely linked to surgical stress response, tissue healing, and infection resistance. PNI has demonstrated prognostic value across various malignancies and surgical settings [14–17]. In ESCC, low PNI is associated with worse overall and recurrence–free survival [18, 19], and recent evidence suggests it may predict short–term complications after nICT [20].

Nevertheless, important knowledge gaps remain: the shape of the relationship between PNI and postoperative complications (linear vs. nonlinear) has not been characterized; optimal PNI thresholds for risk stratification in the nICT setting are undefined; and whether the association of PNI differs by treatment response (particularly pCR) is unexplored, despite evidence that immunonutritional factors may interact with immunotherapy efficacy [21].

To address these gaps, we conducted a retrospective cohort study with three objectives: (1) validate the independent association between PNI and postoperative complications after nICT; (2) characterize the shape of this relationship using restricted cubic spline analysis and identify optimal thresholds; and (3) explore whether the association of PNI differs according to pathological response.

## Methods

### Study design and population

This retrospective cohort study included consecutive patients with locally advanced esophageal squamous cell carcinoma who received neoadjuvant immunochemotherapy followed by radical esophagectomy at Affiliated Hospital of Putian University between January 2020 and December 2025. The study was approved by the Institutional Ethics Committee and conducted in accordance with the Declaration of Helsinki. Given the retrospective nature, the requirement for informed consent was waived.

Inclusion criteria were: (1) histologically confirmed ESCC; (2) clinical stage II–IVA (8th AJCC/UICC TNM staging system); (3) receipt of nICT (at least two cycles of programmed death–1 inhibitor combined with platinum–based doublet chemotherapy); (4) subsequent radical esophagectomy with R0 resection; (5) complete clinical, laboratory, and follow–up data.

**Fig. 1:**
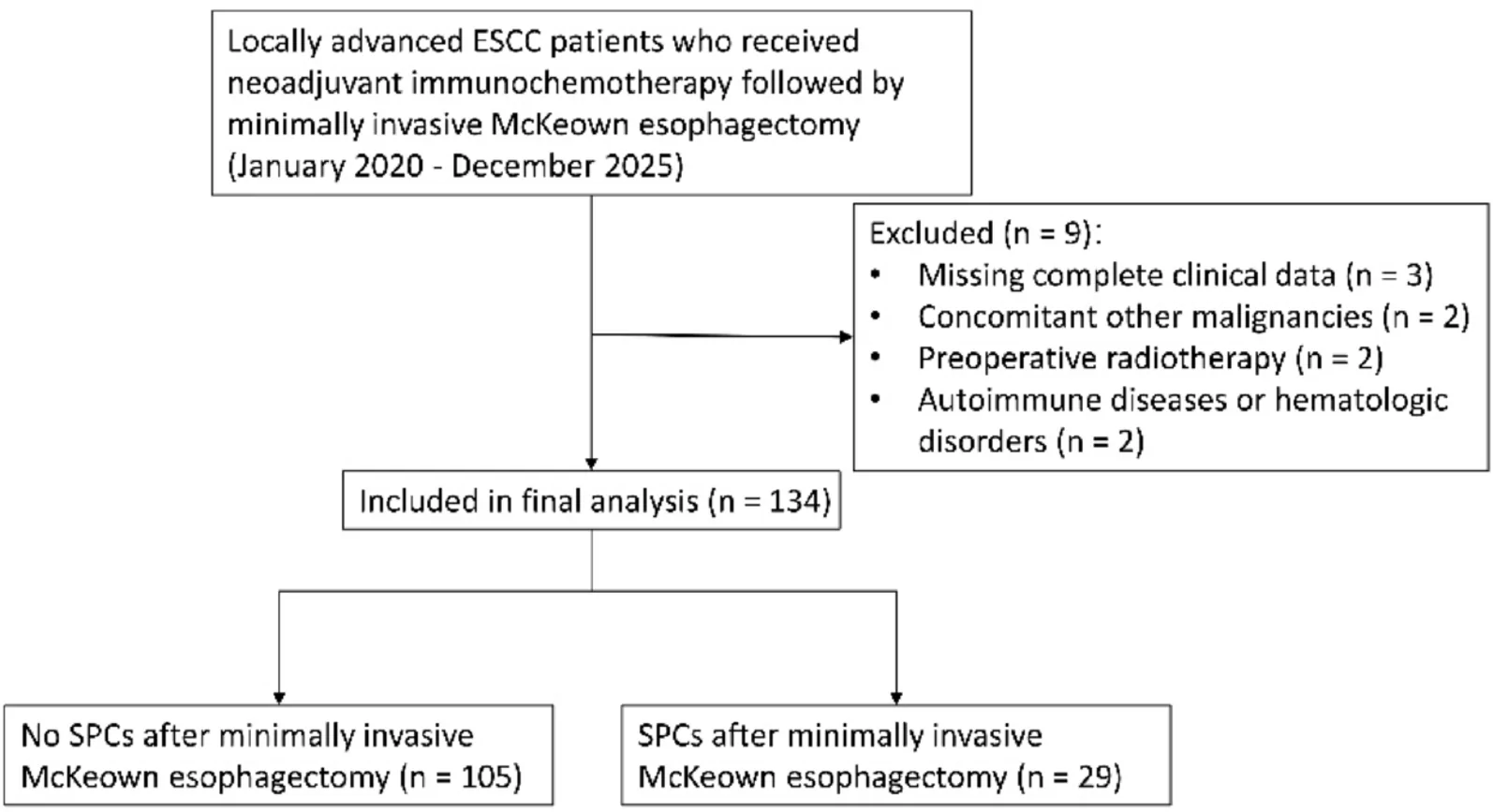
Flowchart of patient selection. A total of 134 patients with locally advanced esophageal squamous cell carcinoma (ESCC) who received neoadjuvant immunochemotherapy followed by minimally invasive McKeown esophagectomy were included in the final analysis. SPCs, severe postoperative complications (Clavien‑Dindo grade ≥ III)

Exclusion criteria were: (1) presence of other synchronous malignancies; (2) preoperative radiotherapy; (3) active infectious, autoimmune, or hematological diseases affecting blood cell counts; (4) emergency surgery or salvage esophagectomy after definitive chemoradiotherapy; (5) missing preoperative laboratory data within one week before surgery. Figure 1 illustrates the patient selection process.

### Neoadjuvant immunochemotherapy and surgical procedure

All patients received at least two cycles of nICT prior to surgery. The regimen consisted of a PD–1 inhibitor (camrelizumab, tislelizumab, pembrolizumab, or nivolumab) combined with platinum–based doublet chemotherapy (cisplatin or carboplatin plus paclitaxel, albumin–bound paclitaxel, or 5–fluorouracil). Clinical response evaluation was performed after completion of nICT using contrast–enhanced computed tomography according to RECIST 1.1 criteria.

Surgery was performed within 4–8 weeks after completion of nICT. All patients underwent radical esophagectomy with the McKeown (three–incision) approach, accompanied by two– or three–field lymphadenectomy as determined by tumor location and surgeon judgment. This procedure was performed using minimally invasive techniques (minimally invasive esophagectomy, MIE). Digestive tract reconstruction was typically performed using a gastric tube via retrosternal or posterior mediastinal route.

### Data collection and definitions

Demographic, clinical, and laboratory data were retrospectively collected from electronic medical records. Preoperative blood samples were obtained within one week before surgery. The prognostic nutritional index was calculated according to Onodera’s formula [13]: PNI = serum albumin (g/L) + 5 × total lymphocyte count (×10⁹/L).

Pathological complete response was defined as the absence of residual viable tumor cells in both the primary tumor and all resected lymph nodes (ypT0N0) according to the 8th AJCC staging system [22]. Tumor regression grade was assessed according to standard criteria; for analytical purposes, TRG 1 was defined as pCR and TRG 2–5 as non–pCR.

Postoperative complications were defined as any complication occurring within 30 days after surgery or during the same hospitalization, including respiratory failure, anastomotic leakage, atrial fibrillation, and others, as diagnosed by attending physicians according to the Esophagectomy Complications Consensus Group (ECCG) definitions [23]. Complications were graded according to the Clavien–Dindo classification [24]. **Severe postoperative complications (SPCs)** were defined as grade III or higher (requiring surgical, endoscopic, or radiological intervention, or leading to organ dysfunction or death). Minor complications (grades I–II) were not included in the primary outcome.

Postoperative respiratory failure was defined as severe impairment of pulmonary ventilation caused by various factors, resulting in inability to maintain adequate gas exchange at rest, manifested as hypoxemia with or without hypercapnia. Under room air conditions, the diagnosis was confirmed by arterial blood gas analysis showing partial pressure of arterial oxygen (PaO₂) < 60 mmHg, with or without partial pressure of arterial carbon dioxide (PaCO₂) > 50 mmHg. The severity of respiratory failure was graded according to the Clavien–Dindo classification based on the required intervention: Grade II: requiring non–invasive ventilation or high–flow nasal cannula; Grade IIIa: requiring reintubation or prolonged invasive mechanical ventilation (> 48 h); Grade IIIb: requiring tracheostomy; Grade IV: life–threatening respiratory failure with single– or multi–organ dysfunction.

### Statistical analysis

Given the exploratory nature of this study and the limited available literature on PNI thresholds in the nICT setting, no formal sample size calculation was performed. The sample size was determined by all eligible patients during the study period. No imputation was performed for missing data; cases with missing values for any covariate were excluded from the corresponding analyses.

All statistical analyses were performed using R software (version 4.1.3, R Foundation for Statistical Computing, Vienna, Austria). Continuous variables were expressed as mean ± standard deviation or median (interquartile range) and compared using Student’s t–test or Mann–Whitney U test as appropriate. Categorical variables were presented as frequencies (percentages) and compared using chi–square test or Fisher’s exact test.

Univariable and multivariable logistic regression analyses were performed to identify factors associated with postoperative complications. Variables with *P* < 0.10 in univariable analysis or those considered clinically relevant were entered into multivariable models. Four models were constructed: Model 1 (unadjusted); Model 2 (minimally adjusted) was adjusted for age and sex; Model 3 (primary parsimonious model adjusted for age, sex, CCI score, and clinical stage (cTNM)); Model 4 A (preoperative model adjusted for age, sex, CCI score, clinical stage, BMI, FEV₁% predicted, and neoadjuvant cycles); Model 4B (perioperative explanatory model adjusted for Model 4 A plus operative time, intraoperative blood loss, and number of lymph nodes dissected). Adjusted odds ratios (ORs) with 95% confidence intervals (CIs) were calculated. Given the limited number of severe postoperative complications (*n* = 29), we used a parsimonious multivariable model (Model 3 (primary parsimonious model)) including only clinically essential pre–specified variables: age, sex, CCI score, clinical stage (cTNM), and PNI. This model contained 5 covariates, yielding an events–per–variable ratio of approximately 5.8, which is within the acceptable range for logistic regression. As a sensitivity analysis, we performed Firth’s penalized logistic regression to address potential overfitting (Supplementary Table 1).

To explore the shape of the relationship between PNI and complication risk, restricted cubic spline regression with three knots (at the 10th, 50th, and 90th percentiles) was fitted within multivariable logistic regression models. Nonlinearity was tested using the Wald test.

Threshold effect analysis was performed using two–piecewise logistic regression models to identify potential inflection points where the relationship between PNI and complications changed. The log–likelihood ratio test compared the one–line linear model with the two–piecewise segmented regression model.

We used a two–piecewise logistic regression model with smoothing to analyze the association threshold between PNI and SPCs. The likelihood–ratio test and the bootstrap resampling method were used to determine inflection points.

Subgroup analyses were conducted according to age, sex, BMI, preoperative stage, neoadjuvant cycles, and pathological response (pCR vs. non–pCR) to assess potential heterogeneity. Interaction terms were tested in multivariable models. Two–sided *P* < 0.05 was considered statistically significant. Subgroup analyses were considered exploratory; adjusted estimates for subgroups with small event counts (e.g., pCR subgroup, *n* = 7 events) are provided in the supplementary material and should be interpreted with caution.

## Results

### Patient characteristics

A total of 134 patients with LA–ESCC who received nICT followed by radical esophagectomy were included in this study. Baseline demographic and clinical characteristics of the 134 patients stratified by the presence of severe postoperative complications (SPCs) are summarized in Table 1. The mean age of the overall cohort was 66.4 ± 8.0 years, and 74.6% were male. Compared with patients without SPCs, those who developed SPCs were significantly older (70.1 ± 7.1 vs. 65.4 ± 8.0 years, *P* = 0.005) and had poorer pulmonary function (FEV₁% predicted: 85.9 ± 22.3 vs. 95.3 ± 14.7, *P* = 0.008). Patients with SPCs also had longer surgery time (6.4 ± 1.5 vs. 5.6 ± 1.1 h, *P* = 0.004), greater intraoperative blood loss (267.2 ± 229.6 vs. 192.9 ± 91.3 mL, *P* = 0.009), and fewer lymph nodes dissected (25.6 ± 12.8 vs. 31.3 ± 12.9, *P* = 0.036). Notably, preoperative PNI was significantly lower in the SPC group (46.9 ± 5.5 vs. 50.8 ± 4.7, *P* < 0.001). No significant differences between the two groups were observed for sex, BMI, smoking history, alcohol history, CCI score, ASA score, neoadjuvant cycles, tumor location, preoperative cTNM stage, hemoglobin, triglycerides, cholesterol, LVEF, pathological stage, or pCR rate.

**Table 1:** Baseline Characteristics of Patients Stratified by Severe Postoperative Complications

Variables	Total (*n* = 134)	SPCs †		*P*–value
		No (*n* = 105)	Yes (*n* = 29)	
**Demographics**				
Age(years), Mean ± SD	66.4 ± 8.0	65.4 ± 8.0	70.1 ± 7.1	0.005
Sex, n (%)				0.256
Male	100 (74.6)	76 (72.4)	24 (82.8)	
Female	34 (25.4)	29 (27.6)	5 (17.2)	
**Clinical Characteristics**				
BMI (kg/m^2^), Mean ± SD	21.9 ± 3.1	21.8 ± 3.2	22.4 ± 2.8	0.302
Smoking history, n (%)				0.733
No	121 (90.3)	94 (89.5)	27 (93.1)	
Yes	13 (9.7)	11 (10.5)	2 (6.9)	
Alcohol history, n (%)				1
No	127 (94.8)	99 (94.3)	28 (96.6)	
Yes	7 (5.2)	6 (5.7)	1 (3.4)	
CCI score, n (%)				1
0/1	118 (88.1)	92 (87.6)	26 (89.7)	
2/3	16 (11.9)	13 (12.4)	3 (10.3)	
ASA score, n (%)				0.246
Ⅰ/Ⅱ	115 (85.8)	92 (87.6)	23 (79.3)	
Ⅲ/Ⅳ	19 (14.2)	13 (12.4)	6 (20.7)	
Neoadjuvant cycles, n (%)				0.221
≤ 3	116 (86.6)	93 (88.6)	23 (79.3)	
>3	18 (13.4)	12 (11.4)	6 (20.7)	
Tumor location, n (%)				0.846
Proximal	85 (63.4)	68 (64.8)	17 (58.6)	
Mid	39 (29.1)	29 (27.6)	10 (34.5)	
Distal	10 (7.5)	8 (7.6)	2 (6.9)	
Preoperative cTNM stage, n (%)				0.448
II	40 (29.9)	33 (31.4)	7 (24.1)	
III	94 (70.1)	72 (68.6)	22 (75.9)	
**Laboratory Parameters**				
Hemoglobin (g/L), Mean ± SD	117.9 ± 15.4	118.3 ± 15.1	116.6 ± 16.4	0.6
Albumin (g/L), Mean ± SD	41.8 ± 3.4	42.4 ± 3.1	39.9 ± 3.7	< 0.001
Lymphocyte count (×10⁹/L), Mean ± SD	1.6 ± 0.6	1.7 ± 0.6	1.4 ± 0.6	0.020
Triglycerides (mmol/L), Median (IQR)	1.2 (0.9, 1.5)	1.2 (0.9, 1.6)	1.1 (0.9, 1.4)	0.197
Cholesterol (mmol/L), Mean ± SD	5.1 ± 1.1	5.1 ± 1.0	5.0 ± 1.5	0.749
FEV₁ (% predicted), Mean ± SD	93.3 ± 17.0	95.3 ± 14.7	85.9 ± 22.3	0.008
LVEF (%), Mean ± SD	64.9 ± 5.4	65.3 ± 5.2	63.7 ± 6.3	0.158
**Preoperative PNI**				
PNI, Mean ± SD	49.9 ± 5.1	50.8 ± 4.7	46.9 ± 5.5	< 0.001
**Surgical and Pathological Data**				
Surgery time(h), Mean ± SD	5.8 ± 1.3	5.6 ± 1.1	6.4 ± 1.5	0.004
Intraoperative blood loss (mL), Mean ± SD	209.0 ± 136.3	192.9 ± 91.3	267.2 ± 229.6	0.009
Lymph nodes dissected (n), Mean ± SD	30.1 ± 13.1	31.3 ± 12.9	25.6 ± 12.8	0.036
Pathological stage (ypTNM), n (%)				0.801
0	39 (29.1)	32 (30.5)	7 (24.1)	
I/II	52 (38.8)	40 (38.1)	12 (41.4)	
III/IV	43 (32.1)	33 (31.4)	10 (34.5)	
Pathological complete response, n (%)				0.506
No	95 (70.9)	73 (69.5)	22 (75.9)	
Yes	39 (29.1)	32 (30.5)	7 (24.1)	

SPCs, severe postoperative complications; SD, standard deviation; IQR, interquartile range; BMI, body mass index; CCI, Charlson Comorbidity Index; ASA, American Society of Anesthesiologists; FEV₁, forced expiratory volume in 1 s; LVEF, left ventricular ejection fraction; PNI, prognostic nutritional index; pCR, pathological complete response

Data are presented as mean ± SD, median (IQR), or number (%). †SPCs were defined as Clavien-Dindo grade ≥ III

Postoperative outcomes are summarized in Table 2. Severe postoperative complications (SPCs) occurred in 29 patients (21.6%). The most frequent SPC was respiratory failure, which developed in 19 patients (14.2% overall; 62.1% of SPC patients). Atrial fibrillation occurred in 25 patients (18.7%), and anastomotic leakage in 13 patients (9.7%). However, among these, only those meeting Clavien–Dindo grade ≥ III criteria were counted as SPCs (see Supplementary Table 2 for grade distribution). Patients with SPCs had significantly longer postoperative hospital stays (24.6 ± 16.4 vs. 18.3 ± 8.7 days, *P* = 0.007) and substantially higher total medical costs (median: 88666.4 vs. 63761.5 CNY, *P* < 0.001) compared to those without SPCs.

**Table 2:** Postoperative outcomes

Outcomes	Total (*n* = 134)	SPCs †		*P*–value
		No (*n* = 105)	Yes (*n* = 29)	
Respiratory failure, n (%)				< 0.001
No	115 (85.8)	104 (99)	11 (37.9)	
Yes	19 (14.2)	1 (1)	18 (62.1)	
Atrial fibrillation, n (%)				0.448
No	109 (81.3)	84 (80)	25 (86.2)	
Yes	25 (18.7)	21 (20)	4 (13.8)	
Anastomotic leakage, n (%)				0.733
No	121 (90.3)	94 (89.5)	27 (93.1)	
Yes	13 (9.7)	11 (10.5)	2 (6.9)	
Postoperative hospital stay (days), Mean ± SD	19.7 ± 11.1	18.3 ± 8.7	24.6 ± 16.4	0.007
Total cost (CNY), Median (IQR)	65515.6 (57566.1, 77503.1)	63761.5 (56159.2, 72035.3)	88666.4 (72913.7, 108626.8)	< 0.001

SPCs, severe postoperative complications (Clavien-Dindo grade ≥ III); SD, standard deviation; IQR, interquartile range; CNY, Chinese Yuan

Data are presented as mean ± SD, median (IQR), or number (%). †SPCs were defined as Clavien-Dindo grade ≥ III. Some patients had multiple complications. One patient in the non-SPC group developed mild respiratory failure (grade II) that did not meet the SPC definition

### Severe postoperative complications

About 21.6% (29 of 134 cases) of patients experienced SPCs postoperatively, including respiratory failure (requiring mechanical ventilation) (*n* = 18; 62.1%), anastomotic leak (requiring surgical intervention) (*n* = 2; 6.9%), atrial fibrillation (hemodynamically significant) (*n* = 4; 13.8%), postoperative hemorrhage (requiring reoperation) (*n* = 1; 3.4%), chylothorax (requiring intervention) (*n* = 1; 3.4%), empyema(requiring drainage) (*n* = 9; 31.0%), and pneumothorax (requiring intervention) (*n* = 2; 6.9%) (Supplementary Table 2).

### Univariate analysis for SPCs

Univariable logistic regression analysis of factors associated with severe postoperative complications (SPCs) is presented in Table 3. Older age (OR = 1.09 per year, 95% CI: 1.02–1.16, *P* = 0.007), lower FEV₁% predicted (OR = 0.97 per %, 95% CI: 0.94–0.99, *P* = 0.011), longer surgery time (OR = 1.60 per hour, 95% CI: 1.14–2.23, *P* = 0.006), greater intraoperative blood loss (OR = 1.04 per 10 mL, 95% CI: 1.00–1.08, *P* = 0.035), and a lower number of lymph nodes dissected (OR = 0.96 per node, 95% CI: 0.93–1.00, *P* = 0.039) were significantly associated with SPCs. Notably, lower preoperative PNI as a continuous variable was strongly associated with increased SPC risk (OR = 0.84 per unit, 95% CI: 0.76–0.93, *P* < 0.001). When PNI was categorized into quartiles, compared with the lowest quartile (Q1, ≤ 46.43), the second (Q2, OR = 0.20, 95% CI: 0.06–0.64, *P* = 0.007), third (Q3, OR = 0.11, 95% CI: 0.03–0.44, *P* = 0.002), and fourth (Q4, OR = 0.19, 95% CI: 0.06–0.62, *P* = 0.006) quartiles all showed significantly reduced risk of SPCs. Other variables, including sex, BMI, smoking, alcohol use, CCI score, hemoglobin, triglycerides, cholesterol, LVEF, ASA score, neoadjuvant cycles, tumor location, preoperative cTNM stage, pathological stage, and pCR status, were not significantly associated with SPCs in univariable analysis (all *P* > 0.05).

**Table 3:** Univariate Logistic Regression Analysis for Severe Postoperative Complications

Variable	OR_95CI	*P*–value
Age (per year)	1.09 (1.02–1.16)	0.007
Sex (Female vs. Male)	0.55 (0.19–1.57)	0.261
BMI (per kg/m²)	1.07 (0.94–1.22)	0.301
Smoking history (Yes vs. No)	0.63 (0.13–3.03)	0.567
Alcohol history (Yes vs. No)	0.59 (0.07–5.1)	0.631
CCI score (2/3 vs. 0/1)	0.82 (0.22–3.08)	0.765
Hemoglobin (per g/L)	0.99 (0.97–1.02)	0.597
Albumin (per g/L)	0.8 (0.7–0.91)	0.001
Lymphocyte count (per 10⁹/L)	0.4 (0.18–0.88)	0.023
Triglycerides (per mmol/L)	0.8 (0.45–1.39)	0.423
Cholesterol (per mmol/L)	0.94 (0.65–1.36)	0.747
FEV₁ (% predicted, per %)	0.97 (0.94–0.99)	0.011
LVEF (%, per %)	0.94 (0.87–1.02)	0.161
ASA score (Ⅲ/Ⅳ vs. Ⅰ/Ⅱ)	1.85 (0.63–5.38)	0.261
Neoadjuvant cycles (>3 vs. ≤3)	2.02 (0.69–5.96)	0.202
Tumor location (vs. Proximal)		
Mid	1.38 (0.56–3.37)	0.481
Distal	1 (0.19–5.15)	0.999
Preoperative cTNM stage (III vs. II)	1.44 (0.56–3.71)	0.449
Surgery time (per hour)	1.6 (1.14–2.23)	0.006
Intraoperative blood loss (per 10 mL)	1.04 (1–1.08)	0.035
Lymph nodes dissected (per node)	0.96 (0.93–1)	0.039
Pathological stage (vs. 0)		
I/II	1.37 (0.48–3.89)	0.552
III/IV	1.39 (0.47–4.08)	0.555
Pathological complete response (No vs. Yes)	1.38 (0.53–3.55)	0.507
**PNI (per unit)**	0.84 (0.76–0.93)	< 0.001
**PNI Quartiles**		
Q1 (Ref.)	1.00	
Q2	0.20 (0.06–0.64)	0.007
Q3	0.11 (0.03–0.44)	0.002
Q4	0.19 (0.06–0.62)	0.006

OR, odds ratio; CI, confidence interval; BMI, body mass index; CCI, Charlson Comorbidity Index; FEV₁, forced expiratory volume in 1 s; LVEF, left ventricular ejection fraction; ASA, American Society of Anesthesiologists; PNI, prognostic nutritional index

Q1 ≤ 46.43, Q2 46.44–50.32, Q3 50.33–53.41, Q4 ≥ 53.42. Data are presented as OR (95% CI). Bold P values indicate statistical significance (*P* < 0.05)

### Multivariable analysis for SPCs

Multivariable logistic regression analyses were performed to evaluate the independent association between PNI and severe postoperative complications (SPCs) across five models with progressively increasing adjustment (Table 4).

**Table 4:** Multivariable Logistic Regression Analyses for the Association Between PNI and Severe Postoperative Complications

Variable	Model 1 (Unadjusted)		Model 2 (Minimally adjusted)		Model 3 (Primary parsimonious)		Model 4 A (Preoperative)		Model 4B (Perioperative explanatory)	
	OR (95%CI)	*P*–value	OR (95%CI)	*P*–value	OR (95%CI)	*P*–value	OR (95%CI)	*P*–value	OR (95%CI)	*P*–value
**PNI (per unit)**	0.84 (0.76–0.93)	< 0.001	0.86 (0.78–0.96)	0.005	0.86 (0.78–0.96)	0.006	0.84 (0.74–0.95)	0.004	0.84 (0.73–0.95)	0.007
**PNI Quartiles**										
Q1 (Ref.)	1.00		1(Ref)		1(Ref)		1(Ref)		1(Ref)	
Q2	0.20 (0.06–0.64)	0.007	0.22 (0.07–0.75)	0.015	0.22 (0.06–0.75)	0.016	0.18 (0.04–0.77)	0.021	0.14 (0.03–0.74)	0.02
Q3	0.11 (0.03–0.44)	0.002	0.11 (0.03–0.46)	0.002	0.11 (0.03–0.46)	0.003	0.07 (0.01–0.37)	0.002	0.05 (0.01–0.31)	0.001
Q4	0.19 (0.06–0.62)	0.006	0.31 (0.09–1.08)	0.066	0.29 (0.08–1.06)	0.061	0.21 (0.05–0.9)	0.035	0.17 (0.04–0.83)	0.028
**P for trend**		0.002	0.57 (0.37–0.89)	0.014	0.57 (0.36–0.9)	0.016	0.53 (0.32–0.87)	0.012	0.49 (0.29–0.84)	0.01

PNI, prognostic nutritional index; OR, odds ratio; CI, confidence interval; Q1 ≤ 46.43, Q2 46.44–50.32, Q3 50.33–53.41, Q4 ≥ 53.42

Model 1: unadjusted. Model 2: adjusted for age and sex. Model 3 (primary parsimonious model): adjusted for age, sex, CCI score, and clinical stage (cTNM). Model 4 A (preoperative model): adjusted for age, sex, CCI score, clinical stage, BMI, FEV₁% predicted, and neoadjuvant cycles. Model 4B (perioperative explanatory model): adjusted for Model 4 A plus operative time, intraoperative blood loss, and number of lymph nodes dissected. Bold P values indicate statistical significance (*P* < 0.05).PNI as a continuous variable and PNI quartiles were analyzed in separate models; they were not included together in any model

When analyzed as a continuous variable, PNI was significantly associated with reduced SPC risk in all models. In the unadjusted model (Model 1), the odds ratio (OR) per unit increase in PNI was 0.84 (95% CI: 0.76–0.93, *P* < 0.001). After adjusting for age and sex (Model 2), the association remained significant (OR = 0.86, 95% CI: 0.78–0.96, *P* = 0.005). In the primary parsimonious model (Model 3, adjusted for age, sex, CCI score, and clinical stage), the OR was 0.86 (95% CI: 0.78–0.96, *P* = 0.006). Further adjustment for BMI, FEV₁% predicted, and neoadjuvant cycles in the preoperative model (Model 4 A) yielded an OR of 0.84 (95% CI: 0.74–0.95, *P* = 0.004). Adding intraoperative variables (operative time, blood loss, and lymph node count) in the perioperative explanatory model (Model 4B) gave an OR of 0.84 (95% CI: 0.73–0.95, *P* = 0.007).

When PNI was analyzed as quartiles (Q1: ≤46.43, Q2: 46.44–50.32, Q3: 50.33–53.41, Q4: ≥53.42), a clear dose–response relationship was observed. In Model 3, compared with Q1, the adjusted ORs for Q2, Q3, and Q4 were 0.22 (95% CI: 0.06–0.75, *P* = 0.016), 0.11 (95% CI: 0.03–0.46, *P* = 0.003), and 0.29 (95% CI: 0.08–1.06, *P* = 0.061), respectively, with a significant trend across quartiles (P for trend = 0.016). Similar patterns were observed in all models, with the strongest protective effect seen in Q3. The trend test was significant across all adjusted models (P for trend ranging from 0.010 to 0.016), confirming a monotonic decrease in SPC risk with increasing PNI category.

These results indicate that higher PNI is independently associated with a lower risk of severe postoperative complications, and the association is robust to adjustment for a wide range of preoperative and intraoperative covariates.

The full results of all multivariable models, including the effect estimates for all covariates, are presented in Supplementary Table 6. In the primary parsimonious model (Model 3), older age remained significantly associated with SPCs (OR = 1.08 per year, 95% CI: 1.00–1.16, *P* = 0.043), while sex, CCI score, and clinical stage did not reach statistical significance. In the preoperative model (Model 4 A), higher BMI showed a significant association with increased SPC risk (OR = 1.24 per kg/m², 95% CI: 1.03–1.49, *P* = 0.020). However, after adding intraoperative variables in the perioperative explanatory model (Model 4B), the effect of BMI became borderline non–significant (OR = 1.21, 95% CI: 1.00–1.46, *P* = 0.051). FEV₁% predicted, neoadjuvant cycles, and intraoperative variables (surgery time, blood loss, lymph node count) were not independently associated with SPCs after full adjustment (all *P* > 0.05). The consistent direction and magnitude of the PNI effect across all models (as described in Table 4) confirm the robustness of its association with SPCs, independent of the other covariates examined.

Given the robustness of the association between PNI and SPCs demonstrated above, we further examined whether the individual components of PNI independently contribute to this association. We repeated the primary parsimonious multivariable analysis (Model 3) replacing PNI with albumin alone, lymphocyte count alone, and both together (Supplementary Table 7). Albumin was significantly associated with SPCs when analyzed alone (OR = 0.82 per g/L, 95% CI: 0.71–0.95, *P* = 0.006) and remained significant after adjusting for lymphocyte count (OR = 0.83, 95% CI: 0.72–0.96, *P* = 0.012). In contrast, lymphocyte count alone showed a non–significant association (OR = 0.53 per 10⁹/L, 95% CI: 0.24–1.18, *P* = 0.119), and its effect became even weaker when albumin was included (OR = 0.62, 95% CI: 0.27–1.45, *P* = 0.273). These results suggest that the protective effect of PNI is largely driven by albumin, although the combined index may capture additional prognostic information.

Although the primary focus of this study was to assess association rather than formal prediction, we explored the predictive performance of PNI as a sensitivity analysis (Supplementary Table 3; Supplementary Figs. 1–3). The area under the ROC curve for PNI was 0.717 (95% CI: 0.596–0.837), with a sensitivity of 72% and a specificity of 76%. The negative predictive value was 91%, indicating that a PNI ≥ 50 effectively identifies patients at low risk of severe complications. Calibration and decision curve analyses showed acceptable model performance. When compared with albumin alone and lymphocyte count alone, PNI showed numerically higher AUC values. These results should be interpreted as exploratory, and external validation is needed.

### Nonlinear relationship and threshold effect analysis

**Fig. 2:**
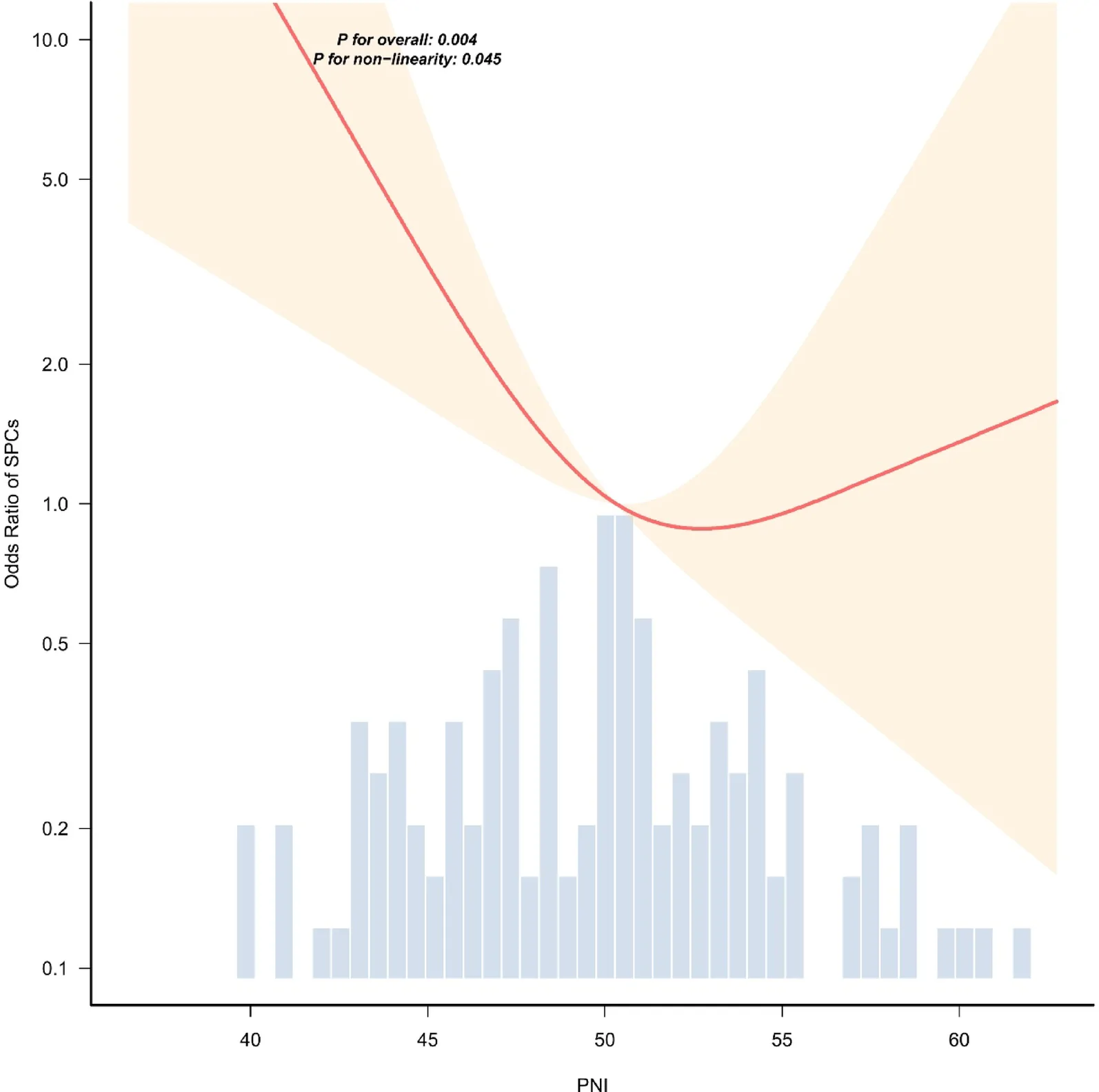
Restricted cubic spline analysis of the association between PNI and severe postoperative complications. The solid red line represents the adjusted odds ratio; the dashed lines indicate the 95% confidence interval. The model was adjusted for age, sex, clinical stage (cTNM), and CCI score. The vertical dashed line marks the inflection point at PNI = 50.00. P for non-linearity = 0.045

Restricted cubic spline analysis revealed a nonlinear “L–shaped” relationship between PNI and SPC risk (P for non–linearity = 0.045) (Fig. 2). The risk of complications decreased progressively with increasing PNI up to a certain point, after which the curve plateaued. This pattern was consistent in both the adjusted analysis (Fig. 2) and the unadjusted analysis (Supplementary Fig. 4).

Threshold effect analysis using two–piecewise logistic regression identified 50.00 as the inflection point (Table 5). When PNI was below 50.00, each one–unit increase was associated with a significant 25% reduction in SPC risk (adjusted OR = 0.75, 95% CI: 0.594–0.936, *P* = 0.011). In contrast, when PNI exceeded 50.00, further increases were not associated with additional risk reduction (adjusted OR = 1.09, 95% CI: 0.872–1.372, *P* = 0.438). The log–likelihood ratio test confirmed that the two–piecewise model provided significantly better fit than the linear model (*P* = 0.023). Among 134 patients, 64 had PNI < 50.00 (24 events, event rate 37.5%) and 70 had PNI ≥ 50.00 (5 events, event rate 7.1%).

**Table 5:** Threshold Effect Analysis of PNI on Severe Postoperative Complications Using a Two–Piecewise Linear Regression Model

PNI Threshold	*N*	Adjusted OR	95%CI	*P* value
<50.00	64	0.75	0.594–0.936	0.011
≥ 50.00	70	1.09	0.872–1.372	0.438
Likelihood Ratio test	–	–	–	0.023

PNI, prognostic nutritional index; OR, odds ratio; CI, confidence interval

The model was adjusted for age, sex, clinical stage (cTNM), and CCI score. The likelihood ratio test compares the two-piecewise model with a linear model

Sensitivity analysis using alternative cutoffs (Supplementary Table 4) confirmed that PNI dichotomized at 50.00 yielded the strongest and most consistent association with SPCs.

### Subgroup analysis

**Fig. 3:**
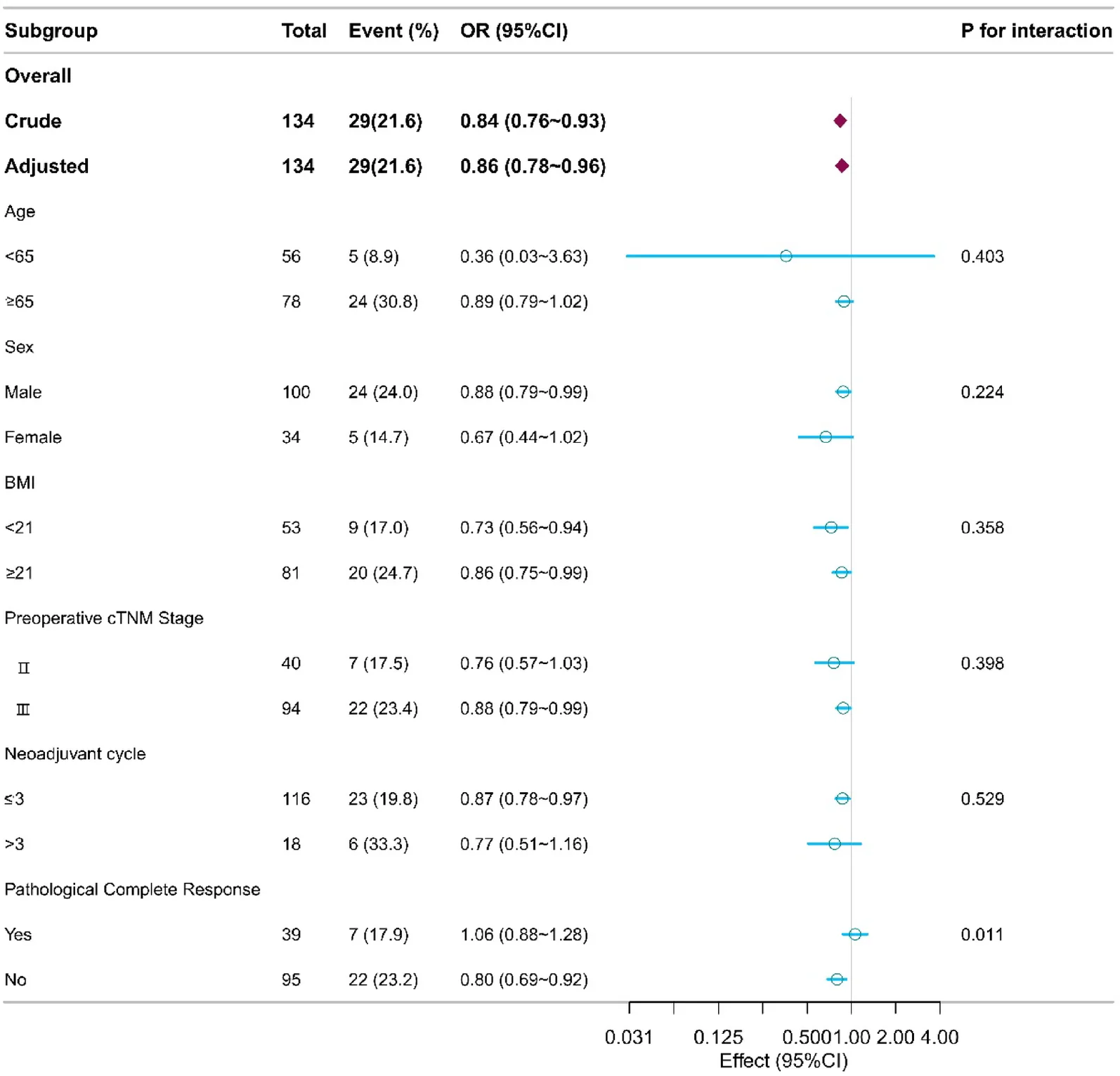
Forest plot of subgroup analysis for the association between PNI and severe postoperative complications. Points represent adjusted odds ratios; horizontal lines indicate 95% confidence intervals. The model was adjusted for age, sex, BMI, FEV₁% predicted, CCI score, neoadjuvant cycles, surgery time, intraoperative blood loss, number of lymph nodes dissected, and pathological complete response (pCR), except for the stratifying variable in each subgroup. The vertical dashed line marks the overall effect estimate. P values for interaction are shown for each subgroup

Subgroup analyses were performed to evaluate the consistency of the PNI–SPCs association across different patient subgroups (Table 6; Fig. 3). The protective effect of higher PNI was consistent across most subgroups, including age, sex, BMI, preoperative stage, and neoadjuvant cycles, with no significant interactions observed (all P for interaction > 0.05).

**Table 6:** Subgroup Analysis for the Association Between PNI and Severe Postoperative Complications

Subgroup	*n*.total	*n*.event_%	OR_95CI	*P*.for.interaction
**Overall**				
**Crude**	134	29(21.6)	0.84 (0.76–0.93)	
**Adjusted**	134	29(21.6)	0.86 (0.78–0.96)	
**Age**				0.233
<65 years	56	5 (8.9)	0.79 (0.62–0.99)	
≥ 65 years	78	24 (30.8)	0.89 (0.79–1.01)	
**Sex**				0.224
Male	100	24 (24.0)	0.88 (0.79–0.99)	
Female	34	5 (14.7)	0.67 (0.44–1.02)	
**BM**I				0.358
<21 kg/m²	53	9 (17.0)	0.73 (0.56–0.94)	
≥ 21 kg/m²	81	20 (24.7)	0.86 (0.75–0.99)	
**Preoperative cTNM Stage**				0.398
Ⅱ	40	7 (17.5)	0.76 (0.57–1.03)	
Ⅲ	94	22 (23.4)	0.88 (0.79–0.99)	
**Neoadjuvant Cycles**				0.529
≤ 3	116	23 (19.8)	0.87 (0.78–0.97)	
>3	18	6 (33.3)	0.77 (0.51–1.16)	
**Pathological Complete Response**				0.011^†^
Yes	39	7 (17.9)	1.06 (0.88–1.28)	
No	95	22 (23.2)	0.8 (0.69–0.92)	

PNI, prognostic nutritional index; OR, odds ratio; CI, confidence interval; BMI, body mass index

Each subgroup analysis was adjusted for all other covariates in Model 3 (primary parsimonious model) except the stratifying variable

†Due to the small number of events in the pCR subgroup (*n* = 7), adjusted odds ratios are presented with a cautionary note about estimate instability and are intended for exploratory purposes only. Crude analyses for this subgroup are shown in Supplementary Table 5. These findings should be considered hypothesis-generating rather than conclusive

However, a significant interaction was observed according to pathological response (P for interaction = 0.002). In patients who did not achieve pCR (*n* = 95), higher PNI was associated with reduced SPC risk (adjusted OR = 0.74, 95% CI: 0.62–0.89). In contrast, among patients who achieved pCR (*n* = 39, 7 events), no significant association was observed (crude OR = 1.11, 95% CI: 0.89–1.39). Due to the small number of events in the pCR subgroup (*n* = 7), the adjusted odds ratios are reported with a cautionary note about estimate instability, and are intended for exploratory purposes only. Crude analyses for this subgroup are presented in Supplementary Table 5. These findings should be considered hypothesis‑generating rather than conclusive.

## Discussion

This retrospective cohort study of 134 patients with locally advanced esophageal squamous cell carcinoma undergoing neoadjuvant immunochemotherapy followed by esophagectomy provides several important findings regarding the relationship between preoperative prognostic nutritional index and severe postoperative complications (SPCs; Clavien–Dindo grade ≥ III). First, we confirmed that PNI is independently associated with postoperative complications, consistent with prior studies in various surgical oncology settings [14–17, 20, 25]. Second, we identified a nonlinear “L–shaped” relationship with a threshold of 50.00, below which complication risk decreased progressively with increasing PNI, but above which further PNI elevation conferred no additional benefit. Third, we observed significant heterogeneity according to pathological response, with the protective effect of higher PNI attenuated in patients achieving pCR.

### Association between PNI and severe postoperative complications

The independent association of PNI with postoperative complications aligns with a growing body of evidence supporting immunonutritional assessment in surgical risk stratification. A recent meta–analysis by Pierce et al. demonstrated that nutritional indices, particularly combined indices like PNI, are strongly associated with postoperative outcomes in upper gastrointestinal malignancies [15]. In the specific context of esophageal cancer, Zou et al. recently reported that preoperative PNI was significantly associated with short–term postoperative complications in 77 ESCC patients after nICT, with a cutoff of 47.38 [20]. Our study extends these findings by demonstrating the persistence of this association after rigorous multivariable adjustment and by characterizing its nonlinear nature.

The biological plausibility of PNI as a marker of surgical outcomes is well–grounded. Serum albumin, a key component of PNI, reflects not only nutritional reserves but also systemic inflammatory status, as pro–inflammatory cytokines downregulate albumin synthesis [26]. Hypoalbuminemia impairs tissue healing, collagen synthesis, and immune competence, predisposing to anastomotic leakage and infectious complications [27]. Lymphocytes, the other component, are central to cell–mediated immunity and defense against pathogens; lymphopenia has been associated with increased susceptibility to postoperative infections [28]. The combination of these two parameters in a single index may capture synergistic effects, explaining why combined indices outperform individual markers in reflecting overall immunonutritional status [15].

To further dissect the contribution of each PNI component, we separately analyzed albumin and lymphocyte count in the primary multivariable model (Supplementary Table 7). Albumin alone was significantly associated with reduced SPC risk (OR = 0.82 per g/L, *P* = 0.006), and its effect remained significant even after adjustment for lymphocyte count (OR = 0.83, *P* = 0.012). In contrast, lymphocyte count alone showed a non–significant association (OR = 0.53, *P* = 0.119), and its effect further weakened when albumin was included. These findings suggest that the protective association of PNI is largely driven by albumin, a marker that integrates both nutritional status and systemic inflammation [26, 27]. Hypoalbuminemia reflects not only poor nutrition but also an ongoing inflammatory response that impairs tissue healing and immune competence [27]. Although lymphocyte count alone was not a significant independent correlate in our cohort—possibly due to the relatively narrow range of lymphocyte values (median 1.6 × 10⁹/L) and the limited number of events—the combined PNI may still offer added value by capturing the synergistic effects of nutritional and immune status. Indeed, previous meta–analyses have shown that composite indices like PNI are more strongly associated with postoperative outcomes than individual markers [15]. Therefore, while albumin appears to be the dominant driver, the integrated PNI remains a practical and robust measure for preoperative risk assessment.

### Threshold effect and clinical implications

The current data suggest a possible nonlinear association, but external validation is needed to confirm PNI = 50 as a definitive clinical cutoff. This value is remarkably consistent with thresholds reported in other populations: Yang et al. identified a similar inflection point of 50.3 for mortality prediction in geriatric hip fracture patients [29], and Chen et al. found that preoperative PNI ≤ 50.9 was an independent risk factor for postoperative delirium after esophagectomy [30]. This convergence across diverse surgical populations and outcomes suggests that 50.00 may represent a biologically meaningful threshold reflecting adequate immunonutritional reserves for surgical stress response.

The “L–shaped” relationship implies that nutritional interventions may yield diminishing returns once patients exceed this threshold. From a clinical perspective, this suggests that preoperative nutritional optimization efforts should prioritize patients with PNI below 50.00, as they have the greatest potential for risk reduction. Conversely, patients with PNI above 50.00 may already possess sufficient immunonutritional reserves, and further aggressive nutritional support may not translate into meaningful complication reduction. This threshold–based approach could facilitate more efficient allocation of limited nutritional support resources.

The identification of PNI = 50.00 as a threshold has direct clinical applications. Preoperatively, patients with PNI < 50.00 should be flagged as high–risk and considered for targeted nutritional support, such as immunonutrition supplementation or early enteral nutrition planning. Intraoperatively, this threshold may inform surgical decision–making, such as the extent of lymphadenectomy or the construction of a protective stoma. Postoperatively, these patients may benefit from enhanced monitoring and early intervention protocols.

### Heterogeneity by pathological response

The observed attenuation of the PNI–complication association in patients achieving pathological complete response (pCR) represents a novel finding. However, because pCR status is not known before surgery, this finding cannot be used to guide preoperative nutritional optimization or perioperative risk stratification. The interaction is presented solely to generate hypotheses for future research.

Several potential explanations merit discussion. First, pCR may serve as a marker of underlying biological factors that also influence surgical outcomes. Patients achieving pCR may have more robust antitumor immune responses, which could reflect generally better immunological competence that independently protects against postoperative complications [31]. Second, the immunomodulatory effects of successful immunotherapy could alter the relationship between preoperative immune markers and postoperative outcomes [32].

Given the small number of events in the pCR subgroup (*n* = 7, 17.9%), these findings are exploratory. The interaction between pCR status and PNI should be regarded as exploratory and hypothesis–generating. Further studies are needed to determine whether treatment response modifies the relationship between immunonutritional status and postoperative recovery. We do not recommend any change in clinical practice based on anticipated pCR status.

### Comparison with previous studies

Our findings both corroborate and extend previous research. The meta–analysis by Li et al. demonstrated that low pretreatment PNI was significantly associated with poorer overall survival in ESCC, with a pooled HR of 1.418 [16]. Xue et al. similarly confirmed the prognostic value of PNI in esophageal cancer through meta–analysis [17]. In the neoadjuvant setting, Takao et al. demonstrated that pretreatment PNI was an independent prognostic factor regardless of neoadjuvant chemotherapy administration, and that patients with improved PNI after neoadjuvant therapy had better outcomes [19]. Our study extends this work by focusing specifically on the nICT era and by providing granular characterization of the dose–response relationship.

The threshold of 50.00 identified in our study is slightly higher than the 47.38 reported by Zou et al. [20] and the 47.1 used in some other studies [33]. These differences may reflect variations in patient populations, treatment protocols, or outcome definitions. Importantly, our use of rigorous nonlinear modeling rather than ROC–derived cutoffs may provide a more accurate representation of the underlying biology.

### Strengths and limitations

This study has several strengths. First, to our knowledge, it is the first to comprehensively characterize the nonlinear relationship between PNI and postoperative complications in the nICT setting using restricted cubic spline analysis and threshold effect modeling. Second, the identification of a specific threshold (50.00) provides clinically actionable information for risk stratification. Third, the exploration of heterogeneity by pCR status generates novel hypotheses regarding the interaction between treatment response and immunonutritional status. Fourth, rigorous multivariable adjustment with three progressively adjusted models supports the robustness of our findings. Fifth, the inclusion of a comprehensive set of covariates, including pulmonary function tests, comorbidity indices, and surgical details, minimizes the risk of residual confounding.

However, several limitations must be acknowledged. First, the retrospective design introduces potential selection bias and limits causal inference. Second, the single–center nature may limit generalizability to other populations and healthcare settings. Third, despite comprehensive adjustment, residual confounding cannot be completely excluded. Fourth, PNI was measured at a single preoperative time point; dynamic changes during neoadjuvant therapy may provide additional prognostic information [19]. Fifth, detailed nutritional support data during the perioperative period were not available, which could influence both PNI values and outcomes. Sixth, the sample size, particularly in the pCR subgroup, limited our ability to perform more detailed stratified analyses. Finally, external validation in independent cohorts is necessary before clinical implementation.

### Future directions

These findings suggest several avenues for future research. First, prospective validation studies with larger sample sizes are needed to confirm the threshold of 50.00 and its utility for risk stratification. Second, randomized controlled trials should evaluate whether targeted nutritional interventions in patients with PNI below 50.00 can effectively reduce postoperative complications. Third, the biological mechanisms underlying the attenuated effect in pCR patients warrant investigation, potentially through integrated analyses of tumor microenvironment, systemic immune markers, and surgical stress response. Fourth, dynamic PNI monitoring during neoadjuvant therapy may identify patients whose immunonutritional status deteriorates, representing another opportunity for intervention. Fifth, combining PNI with other biomarkers (e.g., inflammatory indices, body composition measures) may further enhance predictive accuracy. Sixth, given that respiratory failure accounted for a large proportion of severe postoperative complications in our cohort, future studies with larger sample sizes are warranted to specifically explore whether PNI has a particularly strong predictive value for postoperative respiratory failure. We plan to address this important question in subsequent research. Seventh, direct comparisons of the predictive value of PNI across different neoadjuvant treatment strategies (e.g., neoadjuvant chemotherapy, neoadjuvant chemoradiotherapy, and neoadjuvant immunochemotherapy) are currently lacking. Future studies that include cohorts receiving various neoadjuvant regimens are needed to determine whether the optimal PNI threshold and its predictive strength vary according to the type of preoperative therapy. We plan to address this important question in our subsequent research.

## Conclusions

Preoperative prognostic nutritional index is independently associated with severe postoperative complications (Clavien–Dindo grade ≥ III) in patients with locally advanced esophageal squamous cell carcinoma undergoing neoadjuvant immunochemotherapy followed by esophagectomy. The relationship is nonlinear and “L–shaped,” with a threshold of 50.00 below which complication risk decreases progressively with increasing PNI, and above which the risk plateaus. The protective effect of higher PNI is attenuated in patients achieving pathological complete response, highlighting potential interactions between treatment response and immunonutritional status that merit further investigation. These findings provide a clinically actionable threshold for perioperative risk stratification and support personalized nutritional interventions in this high–risk population.

## Data Availability

The datasets generated and/or analyzed during the current study are available from the corresponding author on reasonable request.
